# Existing Funding Sources in Degenerative Cervical Myelopathy Research: Scoping Review

**DOI:** 10.2196/36194

**Published:** 2022-06-30

**Authors:** Henry Bestwick, Jye Quan Teh, Oliver Mowforth, Ben Grodzinski, Mark Kotter, Benjamin Davies

**Affiliations:** 1 Clinical School University of Cambridge Cambridge United Kingdom; 2 Academic Neurosurgery Unit Department of Clinical Neurosurgery University of Cambridge Cambridge United Kingdom

**Keywords:** cervical cord, myelopathy, spondylosis, stenosis, disc herniation, ossification posterior longitudinal ligament, degeneration, research funding, systematic review, spinal cord, patient and public involvement

## Abstract

**Background:**

Degenerative cervical myelopathy (DCM) is a common, disabling condition of symptomatic cervical spinal cord compression that requires significant research advances to improve patient outcomes. A James Lind Alliance Partnership recently identified the top research priorities for DCM. To effectively address these priorities, appropriate funding of DCM research is essential.

**Objective:**

The aim of this paper is to review current funding in DCM research and highlight future research funding opportunities.

**Methods:**

A systematic search of Web of Science for “cervical AND myelopathy” was conducted. Papers exclusively studying DCM with declared funding and published between January 1, 1995, and March 21, 2020, were considered eligible. Funding sources were classified by country of origin and organization type. A grant search was also conducted using Dimensions.ai (Digital Science Ltd).

**Results:**

A total of 621 papers were included, with 300 unique funding bodies. The top funders were AO Spine (n=87); National Institutes of Health, USA (n=63); and National Natural Science Foundation, China (n=63). Funding sources in the USA (n=242) supported the most DCM research, followed by China (n=209) and Japan (n=116). Funding in the USA was primarily provided by corporate or nonprofit organizations (146/242, 60.3%), while in China, the majority of funding was from institutions (208/209, 99.5%). Dimensions.ai gives an estimate for the total declared grant funding awards for DCM-specific research. Data here showed 180 grants awarded specifically for DCM research, with a total value of US $45.6 million since 1996.

**Conclusions:**

DCM funding appears to be predominantly from the USA, China, and Japan, aligning with areas of high DCM research activity and underpinning the importance of funding to increasing research capacity. The existing funding sources differ from medical research in general, representing opportunities for future investment in DCM.

## Introduction

Degenerative cervical myelopathy (DCM), often previously referred to as cervical spondylotic myelopathy, is a progressive, slow motion, spinal cord injury caused by degenerative changes that lead to narrowing of the spinal canal [1]. It is the most common nontraumatic cause of spinal cord impairment [2], with recent estimates suggesting that as many as 1 in 50 adults could be affected in their lifetime [3,4].

DCM can cause a range of symptoms, including loss of manual dexterity, imbalance and falls, and incontinence and pain [1]. The mainstay of treatment is decompressive surgery [5]. Although this has been demonstrated to offer the most meaningful benefit, recovery is rarely complete and most people are left with life-long disabilities [1,6]. In a recent comparison of quality of life in chronic disease, people with DCM were found to have one of the lowest 36-Item Short Form Survey (SF-36) scores of any chronic disease [6]. Research leading to improved outcomes is urgently required.

To formally address this problem, a consensus initiative was established to improve research efficiency in DCM. AO Spine Research Objectives and Common Data Elements for DCM (RECODE-DCM) is an international, multistakeholder partnership between surgeons, health care professionals, and patients [7]. A National Institute for Health Research (NIHR) James Lind Alliance priority setting partnership established the top 10 DCM research priorities, including raising awareness, developing new treatments and diagnostic tools, and acquiring a better understanding of pathophysiology [8].

To enable these questions to be addressed, research funding targeting these priorities is urgently needed. The main aim of this study is to characterize the funding of existing DCM research and identify potential future funding organizations. Within this, our objectives are as follows: to characterize which countries, organizations, and type of organizations fund the majority of DCM research and to provide an overview of the estimated total grant funding in DCM.

## Methods 

To characterize the funding of existing DCM research, we used 2 methods. The first was most closely aligned with a scoping review and involved formulating a research question, identifying relevant studies, and further categorizing and analyzing the results as is standard in a scoping review methodology [9].

### Identifying the Research Question

The aim of this study was to characterize the funding of existing DCM research. Although reviews may normally examine the results of individual papers, our search was focused on extracting the funding information of papers and focusing only on whether the article was on DCM.

To estimate the number of funders in DCM research, we attempted to extract funding details from literature databases. Of the existing medical literature databases, the only database allowing extraction of funding details is Web of Science [10,11].

### Identifying Relevant Studies

There are many different terms used for DCM around the world [12]. Therefore, to ensure the search was comprehensive, the search terms “cervical AND myelopathy” were used. All papers from January 1, 1995, to March 21, 2020, were eligible for inclusion. These were then filtered by the presence of funder details to exclude entries without explicit funding sources.

### Study Selection

The search output was uploaded to Rayyan, a systematic review web platform [13]. Titles and abstracts were then screened independently by 2 authors (JQT and HB). The inclusion criteria for literature were the following: all languages, primary research and systematic or narrative reviews, preclinical and clinical studies, and DCM-related spinal conditions. Meanwhile, the exclusion criteria were the following: corrections, letters, editorials, commentaries, proposals, technical notes, and conference papers; myelopathy not caused by DCM; cervical spinal surgery not specific to DCM; radiculopathy only; and thoracic or lumbar myelopathy. Any conflicts or undecided papers were resolved by discussion between JQT and HB until consensus was reached.

### Estimating DCM Grants Using Dimensions.ai

To supplement our study, we undertook an additional search.

Using a grant-searching function on Dimensions.ai (Digital Science Ltd), we gathered funding information from 1996 to the present day using keywords for DCM [14]. Dimensions.ai is a platform that can be used to search grants awarded for specific research. Dimensions.ai provides information on the research title and abstract, investigator, funding amount, and over what period the research is expected to be completed. However, it does not include information regarding whether an author has been supported by a general scholarship and undertaken research in a given area, nor does it give information if a grant awarded for another project coincidentally funded research in another field. It therefore gives an overview of estimated value for total grant funding awards specifically requested for DCM research alone.

The grant information we gathered included the total number of grants on Dimensions.ai, total grant funding, average grant awarded, and date and amount of earliest grant shown on Dimensions.ai.

As DCM has only recently been proposed as an umbrella term [12], a search was completed using the following DCM-related terms: “degenerative cervical myelopathy,” “cervical spondylotic myelopathy,” “ossification posterior longitudinal ligament,” “ossification ligamentum flavum,” “cervical myelopathy,” “cervical,” and “myelopathy” [15,16]. The search results were then screened manually to identify those specific to DCM. Any irrelevant research was excluded.

### Charting the Results

Typical paper-specific information that could be recorded in a scoping review (such as aims, methodology, and results) was not necessary to answer our question on the sources of funding for DCM research. As the aim was to examine the funding landscape of DCM research, more information regarding the characteristics of funding organizations was required.

### Collating and Summarizing the Results

The total number of papers with a funding body was recorded. These funding bodies were then ordered with regard to how many papers they supported. The funding bodies were further delineated into their country of origin and the type of sector.

Funders were classified by country of origin by 2 authors (JQT and HB). Identifying countries associated with each funder required criteria to classify a funder: the funding body had to be a university, national funding body, provincial or state funding body, or organization or company; the funding body needed to have headquarters in a specific country; and the funding body could not have a country of origin that was unclear.

Some organizations are international without specific association with any particular country and were labeled as such, for example AO Spine. AO Spine is a global organization with headquarters in Switzerland. It is funded by the AO Foundation, with funding distributed globally. It was therefore felt to be best defined as an organization without a specific country of origin. Entries that did not satisfy the criteria were labeled as “unclear.”

To further investigate funders, we classified them into 2 categories: institutional and corporate/charitable. The corporate/charitable group was further classified into for-profit and not-for-profit organizations.

Funders were classified as institutional if they were any of the following: a regional or central governmental funding body, a university, a research institution, or a hospital associated with a university or research institution.

Alternatively, they were classified as corporate/charitable if they were any of the following: a charitable or not-for profit organization or a for-profit organization or corporation.

The number of institutional organizations compared to corporate/charitable organizations was compared on a global and country level. If the funder did not satisfy either set of criteria or if it was unclear which category they would fit into, they were labeled “unclear.”

### Data Analysis

Data cleaning and visualization was conducted using Python [17-19].

## Results

### Study Selection

**Figure 1:**
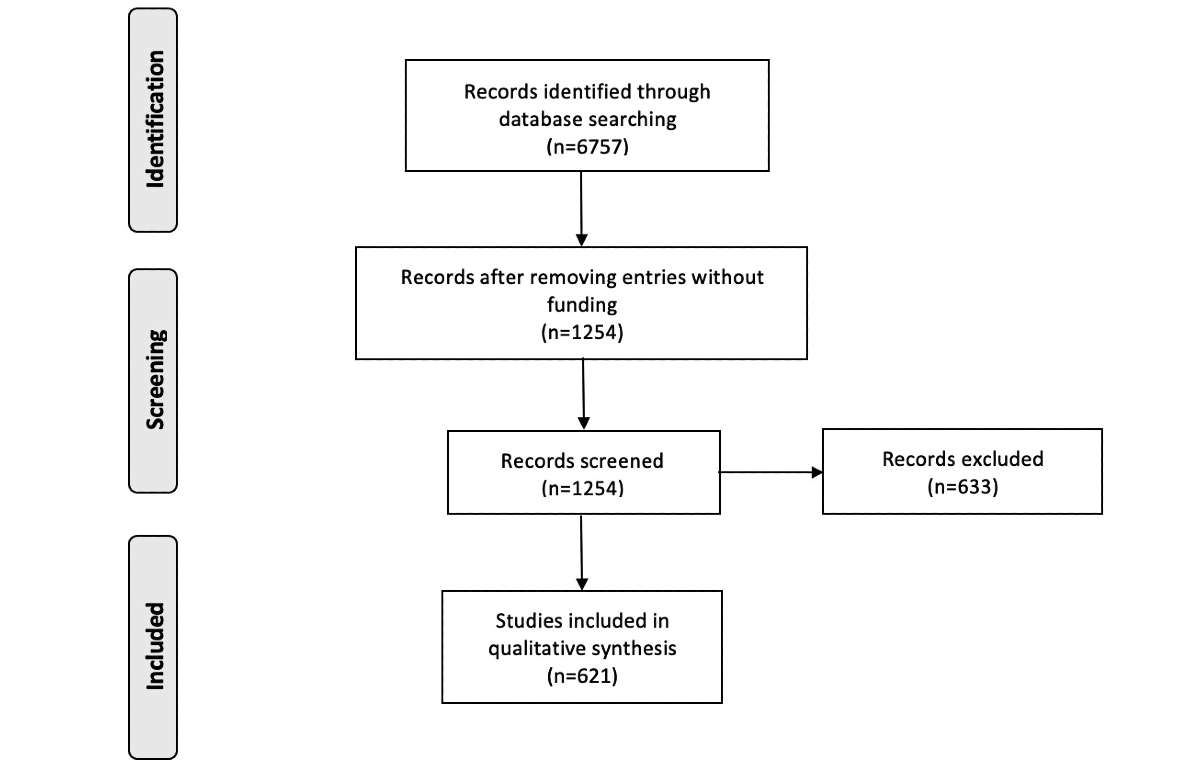
PRISMA (Preferred Reporting Items for Systematic Reviews and Meta-Analyses) flow diagram.

Of the 6757 papers returned from Web of Science, 621 papers acknowledged funding and survived passed application of our inclusion and exclusion criteria (Figure 1).

We identified 300 unique funding bodies that supported DCM research (Table 1). Many research papers had more than 1 funding body: there were a total of 920 references of funding from 300 unique funders.

**Table 1:** Top 10 funding organizations for health research (annual figure, 2013) and DCM-specific research funding.

Rank	Top 10 funding organizations for all health research expenditure, millions^a^	Top 10 funders for DCM^b^-specific research by research output, number of papers
1	National Institutes of Health, 26,081.3	AO Spine, 87
2	European Commission, 3717.7	National Institutes of Health, 63
3	UK Medical Research Council, 1321.5	National Natural Science Foundation of China, 63
4	Institut national de la santé et de la recherche médicale, 1041.2	Ministry of Health, Labour and Welfare Japan, 47
5	United States Department of Defense, 1017.7	Ministry of Education, Culture, Sports, Science and Technology Japan, 25
6	Wellcome Trust, 909.1	DePuy Synthes, 22
7	Canadian Institutes of Health Research, 883.6	Cervical Spine Research Society, 18
8	Australian National Health and Medical Research Council, 777.6	DeZwirek Family Foundation, 18
9	Howard Hughes Medical Institute, 752.0	Gerald and Tootsie Halbert Chair in Neural Repair and Regeneration, 18
10	Deutsche Forschungsgemeinschaft/German Research Foundation, 630.6	National Research Foundation of Korea, 16

^a^In US dollars.

^b^DCM: degenerative cervical myelopathy.

### Top Funders for DCM Research

The 300 funding bodies were then ordered according to the number of papers they supported. Table 1 shows this data for the top 10 DCM-specific funding organizations by research output and compares it to the top 10 funding organizations for general health research.

### Funded DCM Research by Country

**Figure 2:**
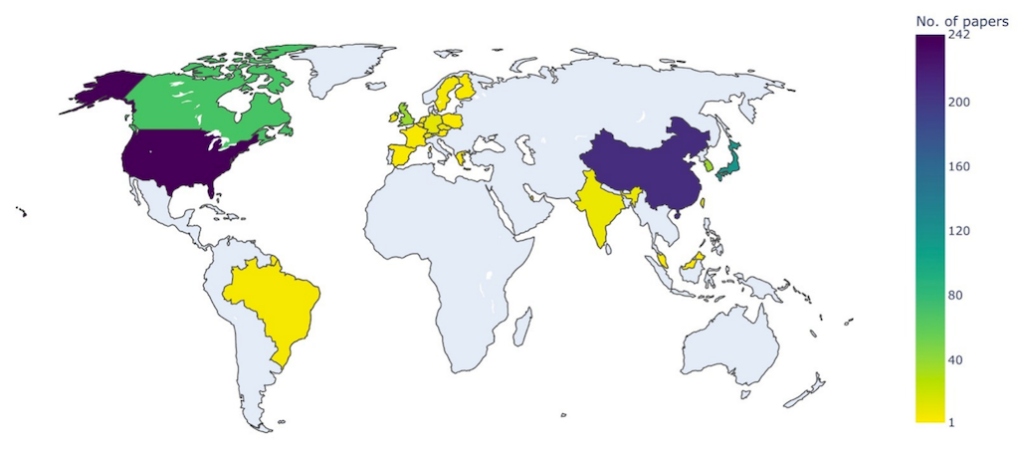
World heat map of degenerative cervical myelopathy funding sources. This map excludes funders that were not clearly associated with a specific country. The greater the number of funders of degenerative cervical myelopathy research, the hotter the colour of the country.

DCM research funding had a global distribution (Figure 2). The top 3 countries for number of funded DCM papers were the United States, China, and Japan, followed by Canada and the United Kingdom (Table 2 and Table 3). There were 112 papers without a specific country of origin, including 87 funded by AO Spine.

**Table 2:** Top 10 funding countries for health research and DCM-specific research funding.

Rank	Top 10 countries for research and development expenditure as a percentage^a^ of that country’s GDP^b^	Top 10 countries for DCM^c^-specific research funding, number of papers
1	Israel, 4.95	USA, 242
2	South Korea, 4.81	China, 209
3	Switzerland, 3.37	Japan, 116
4	Sweden, 3.34	Canada, 69
5	Japan, 3.26	United Kingdom, 38
6	Austria, 3.17	South Korea, 37
7	Germany, 3.09	Hong Kong, 18
8	Denmark, 3.06	Germany, 11
9	United States, 2.84	Switzerland, 8
10	Belgium, 2.82	Ireland, 7

^a^Total values not available.

^b^GDP: gross domestic product.

^c^DCM: degenerative cervical myelopathy.

**Table 3:** Top 10 countries by number of DCM papers that received research funding. The minimum percentage of papers from each country that was supported by research funding is estimated with reference to the total number DCM papers published during this time period from each country [20]. Raw data were requested directly from the author.

Country	Number of papers supported by funding as the percentage of total DCM^a^ papers from the country, n/N (%)
United States	242/314 (77.1)
China	209/409 (51.1)
Japan	116/633 (18.3)
Canada	69/136 (50.7)
United Kingdom	38/60 (63.3)
South Korea	37/122 (30.3)
Hong Kong	18^b^
Germany	11/82 (13.4)
Switzerland	8/17 (47.0)
Ireland	7/9 (77.8)

^a^DCM: degenerative cervical myelopathy.

^b^Full data unavailable.

### Funder Sectors

**Figure 3:**
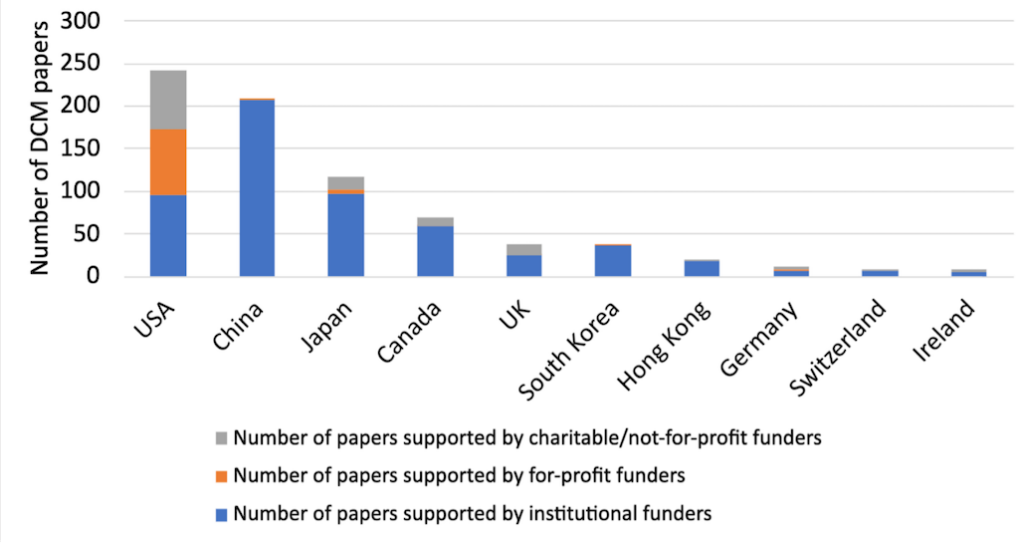
Breakdown of funded papers in the top 10 countries. DCM: degenerative cervical myelopathy.

In total, 598/920 (65%) funding sources were institutional, 318/920 (34.6%) were a corporate/charitable source, and 4/920 (0.4%) were unclear. Of the 318 corporate/charitable sources, 229/318 (72%) were not-for-profit or charitable organizations, and 89/318 (28%) were for-profit corporations. Many funders supported more than 1 paper. The proportion of research funding from institutional and corporate or charitable funders varied across countries (Figure 3). China was the country with the greatest number of papers funded by institutional sources (n=208), followed by Japan (n=96) and the United States (n=96; Table 4). The Unites States was the country with the greatest number of papers funded by corporate or charitable funders (n=146), followed by Japan (n=20) and the United Kingdom (n=14).

**Table 4:** Top 5 countries for papers supported by institutional and corporate/charitable funders.

Funder by country		Portion supported by funder, n/N (%)
**Institutional funders**		
	China	208/209 (99.5)
	Japan	96/116 (82.8)
	United States	96/242 (39.7)
	Canada	58/69 (84.1)
	South Korea	36/37 (97.3)
**Corporate/charitable** **funders**		
	United States	146/242 (60.3)
	Japan	20/116 (17.2)
	United Kingdom	14/38 (36.8)
	Canada	11/69 (15.9)
	Germany	5/11 (45.5)

### Grant Funding Awards

The second part of the results relate to the search on Dimensions.ai. A total of US $45.6 million in grant funding for DCM papers was identified in Dimensions.ai (Table 5). Different results were obtained using variations of the search terms, including differing numbers of grants and a different percentage of grants that were manually verified to be DCM-specific.

**Table 5:** Portion grants that were found to be DCM-specific following manual verification by search term.

Search terms	DCM^a^-specific grants, n/N (%)	Total DCM funding, millions^b^
Degenerative cervical myelopathy	24/24 (100)	11.4
Cervical spondylotic myelopathy	48/63 (76)	14.1
Cervical myelopathy	104/128 (81)	20.0
Ossification posterior longitudinal ligament	4/81 (5)	0.137
Ossification ligamentum flavum	0/20 (0)	0

^a^DCM: degenerative cervical myelopathy.

^b^In US dollars.

## Discussion

### Principal Results

Our study identified 300 unique funding bodies for DCM research. A total of 621 papers acknowledged funding, largely provided by 4 organizations. These, aside from AO Spine, are associated with Japan, China, and the USA. Funding bodies originate from the corporate, charitable, and institutional sectors, but these are distributed unequally across different countries, and research is primarily supported by institutional bodies (598/920, 65%). Our Dimensions.ai research showed a minimum of US $45.6 million dollars of grant funding awards specifically for DCM from 1974 to 2020.

### DCM Research Has Relied On a Small Number of Funders

Viergever and Hendriks [21] identified the top 10 funders for health research globally. They identified the US National Institutes of Health (NIH) as the largest funder of all, but many other leading providers were unrepresented in our DCM review, such as the European Commission, UK Medical Research Council, and the Wellcome Trust, the largest philanthropic funding body for health research [21]. Furthermore, only 5 out of 10 countries in our list of the top 10 countries that support DCM research were included in the top 10 of the World Bank’s 2018 analysis of research and development research expenditure as a percentage of gross domestic product [22]. These countries include the USA, Japan, South Korea, Germany, and Switzerland. Interestingly, while Israel spent the most on research and development per gross domestic product, our analysis did not identify any funding bodies from Israel. Taken together, this suggests many unused funders and identifies opportunities for DCM research.

### Corporate and Charity Sectors Are Underrepresented in DCM

DCM research has relied on institutional organizations, constituting 598 (64.1%) of the listed 920 reported funding sources. Although there are exceptions [11], this contrasts research funding as a whole, which is estimated to be 60% corporate, 30% institutional, and 10% from non-profit organizations [23]. For DCM, the corporate or non-profit sectors remain a challenge. The existing corporate sector for DCM is focused on medical devices and may not be best placed to support the full breadth of research priorities identified by AO Spine RECODE-DCM. For example, DePuy Synthes (a subsidiary of the Johnson & Johnson family of companies) funded 22/621 (3.5%) papers and are solely an orthopedic and neurosurgical device company [24].

Furthermore, while AO Spine has been a notable supporter, DCM does not have a specific funding organization comparable to ones like the Motor Neurone Disease Association or the Multiple Sclerosis Society [25,26]. Charitable organizations are not just significant research funders: they are essential for advocacy. In the United Kingdom for example, the charitable sector contributes £1.6 billion (US $1.7 billion) to medical research [27] and also acts as a lobby group [28]. With Myelopathy.org, DCM now has a dedicated charitable organization, with medical research funding being among its charitable aims [29].

Stakeholders in AO Spine RECODE-DCM have been cognizant of these challenges, which is reflected in the raising of awareness being established as the leading research priority and with understanding the disease burden and socioeconomic impact being among the other priorities.

Begum et al [11], however, demonstrated that burden of disease has been a relatively unimportant driver of research investment or activity. In an analysis of research funding by the US NIH, disease burden correlated poorly with research investment [30]. Instead, funding decisions may be more significantly informed by political influences, public interest, and transmissibility risk [31]. In oncological research, there is a relative paucity of research output for certain cancers, such as lung, esophageal, and pancreatic cancers, despite their increasing burden and poor prognosis [32]. This reinforces the importance of raising awareness for DCM to facilitate funding for research.

### Comparison to Prior Work

In a comparison of these results to a study capturing all DCM papers published in the past 25 years [20], our data suggests that at least 27.46% (621/2261) of DCM research has specific funding.

The location of funding aligns with the location of DCM research output, which has been dominated by the USA, China, Japan, and Canada over the past 20 years [33,34]. This was expected and in keeping with other global health care research investment [10]. It highlights the importance of securing investment to accelerate advances in research outcomes. This is, therefore, now a critical part of ensuring that the aims of the AO Spine RECODE-DCM research priorities are met.

### The Global Context

We identified a lack of DCM research funding originating in many low-and-middle-income countries (LMICs), including no funding from the entire African continent. This is common to many health care fields [23]. Yusuf et al [35] identified potential causes of lack of neuroscience research in Africa, among which insufficient funding was one. This is notable for 2 reasons. First, DCM is a global problem [1,36]. Spinal cord disorders such as DCM will increase with a globally aging population, and the prevalence and mortality of spinal disorders, particularly the cervical spine, are increasing in LMICs [37]. Second, from a funding perspective, there is increasing investment in health care research and development in LMICs [38]. Notable examples include organizations such as the NIH [39], NIHR [40], and Canadian Institutes of Health Research (CIHR) [41], as well as philanthropic organizations, such as the Bill and Melinda Gates Foundation. Although much funding is targeted for specific priorities or diseases, much is also investigator-led. The driving force and overall aims behind this increased global investment is multifaceted [42-44] but nevertheless represents an opportunity for DCM.

### Maximizing Investment and Future Directions

Despite relatively little investment, DCM research has made significant progress, with the number of published papers increasing year on year and many conducted without research funding [33]. This has contributed to many advances in DCM research [45]. This also highlights the fact that investment and research activity may not always be a linear relationship. For example, in a review of global research activity within esophageal cancer, it was identified that the USA published relatively little compared to their overall research expenditures, while Japan published relatively more [10].

This calls for reduced system inefficiencies to maximize the return of research investment [46], for example, by ensuring research aligns with community needs [47] and is conducted in a robust and transparent manner [48,49] such that its findings can be effectively used. Addressing inefficiencies is the aim of AO Spine RECODE-DCM [50-54]. In addition to setting research priorities, it has agreed to a standardized definition and name for the condition and for a minimum data set to be measured in all research studies [7,52,53,55].

### Limitations

There were some limitations to this review. First, information on the funding of DCM research was extracted from the funding metadata in a single research paper database and the acknowledgement sections of published articles therein. Lack of funding information in other common databases prohibited their use. Nonetheless, a database of research funding grants was searched in parallel and the data considered together [56,57]. Although this approach was innovative, systematic, and able to identify a significant amount of data, it is unlikely to have been comprehensive, thus representing a minimum estimate of funding. Research on funding is a largely unexplored area, and the systems in place to document funding sources and tools to support interrogating these systems remain limited and inconsistent.

Second, the funding of published research papers is only a surrogate for research investment [58,59]: it does not quantify the specific amount or role of funding, nor does it account for unpublished research. Moreover, the discrepancies we identified in the results of similar search terms in Dimensions.ai highlights the inconsistency in terminology in this field. However, using papers gives the general overview that our study aims to provide and is a useful and pragmatic way to understand how research is broadly supported.

Third, we categorized funders into institutional, corporate, or charitable groups. However, this may be too simplistic. In reality, organizations are complex and interconnected, with institutions receiving charitable funding [60] and charities receiving corporate backing [28]. Despite this, our study does give a broad understanding over how DCM research is supported by these sectors.

Finally, we note that our review contained studies mainly in English, 1 in German, but none in Chinese. The contribution of Chinese-language papers to global research is significant; Xie and Freeman [61] attribute 37% of global citations in scientific articles to China, compared to our 34% of papers with a Chinese funding origin.

Chinese language papers were not explicitly excluded by our review, but none were identified in the results. We note that Web of Science, our required platform due to its unique ability to extract funding information, searches a relatively small population of Chinese-language papers [61]. There may be a population of DCM papers with a funder originating in China and written in Chinese which has not been included in this study. Although we might have underestimated the total contribution of Chinese funding to DCM research, we still show a substantial contribution. Thus, our study provides as useful, pragmatic, and comprehensive snapshot as is currently feasible.

### Conclusions

This is the first review to attempt a global synthesis of the funding landscape of global DCM research, which highlights opportunities for future DCM research. AO Spine has been the leading funder of DCM research, while on a country-specific basis, DCM research has predominantly been funded by the USA, China, and Japan. As this aligns with areas of high research output, it reaffirms the importance of research investment for accelerating advances in DCM. The paucity of investment from major funding organizations and countries with leading research and development expenditure, alongside the increasing investment in global health research, represents opportunities for DCM.

## Abbreviations

CIHR: Canadian Institutes of Health Research
DCM: degenerative cervical myelopathy
LMIC: low-and-middle-income country
NIH: National Institutes of Health
NIHR: National Institute for Health Research
RECODE-DCM: Research Objectives and Common Data Elements for Degenerative Cervical Myelopathy
SF-36: 36-Item Short Form Survey

## Data Availability

This article uses data available on Web of Science and Dimensions.ai.

## References

[1] Davies BM, Mowforth OD, Smith EK, Kotter MR (2018). Degenerative cervical myelopathy. BMJ.

[2] Kalsi-Ryan S, Karadimas SK, Fehlings MG (2013). Cervical spondylotic myelopathy: the clinical phenomenon and the current pathobiology of an increasingly prevalent and devastating disorder. Neuroscientist.

[3] Kovalova I, Kerkovsky M, Kadanka Z, Nemec Martin, Jurova Barbora, Dusek Ladislav, Jarkovsky Jiri, Bednarik Josef, Kadanka (2016). Prevalence and Imaging Characteristics of Nonmyelopathic and Myelopathic Spondylotic Cervical Cord Compression. Spine (Phila Pa 1976).

[4] Smith Sam S, Stewart Max E, Davies Benjamin M, Kotter Mark R N (2021). The Prevalence of Asymptomatic and Symptomatic Spinal Cord Compression on Magnetic Resonance Imaging: A Systematic Review and Meta-analysis. Global Spine J.

[5] Fehlings MG, Tetreault LA, Riew KD, Middleton JW, Aarabi B, Arnold PM, Brodke DS, Burns AS, Carette S, Chen R, Chiba K, Dettori JR, Furlan JC, Harrop JS, Holly LT, Kalsi-Ryan S, Kotter M, Kwon BK, Martin AR, Milligan J, Nakashima H, Nagoshi N, Rhee J, Singh A, Skelly AC, Sodhi S, Wilson JR, Yee A, Wang JC (2017). A Clinical Practice Guideline for the Management of Patients With Degenerative Cervical Myelopathy: Recommendations for Patients With Mild, Moderate, and Severe Disease and Nonmyelopathic Patients With Evidence of Cord Compression. Global Spine J.

[6] Oh T, Lafage R, Lafage V, Protopsaltis T, Challier V, Shaffrey C, Kim HJ, Arnold P, Chapman J, Schwab F, Massicotte E, Yoon T, Bess S, Fehlings M, Smith J, Ames C (2017). Comparing Quality of Life in Cervical Spondylotic Myelopathy with Other Chronic Debilitating Diseases Using the Short Form Survey 36-Health Survey. World Neurosurg.

[7] Davies BM, Khan DZ, Mowforth OD, McNair AGK, Gronlund T, Kolias AG, Tetreault L, Starkey ML, Sadler I, Sarewitz E, Houlton D, Carter J, Kalsi-Ryan S, Aarabi B, Kwon BK, Kurpad SN, Harrop J, Wilson JR, Grossman R, Curt A, Fehlings MG, Kotter MRN (2019). RE-CODE DCM (search Objectives and ommon ata lements for egenerative ervical yelopathy): A Consensus Process to Improve Research Efficiency in DCM, Through Establishment of a Standardized Dataset for Clinical Research and the Definition of the Research Priorities. Global Spine J.

[8] Mowforth OD, Starkey ML, Kotter MR, Davies BM (2020). Letter to the Editor. The need for research prioritization in cervical myelopathy. J Neurosurg Spine.

[9] Arksey H, O'Malley L (2005). Scoping studies: towards a methodological framework. International Journal of Social Research Methodology.

[10] Klingelhöfer D, Zhu Y, Braun M, Brüggmann D, Schöffel N, Groneberg DA (2019). A world map of esophagus cancer research: a critical accounting. J Transl Med.

[11] Begum M, Lewison G, Sommariva S, Ciani O, Tarricone R, Sullivan R (2017). European diabetes research and its funding, 2002-2013. Diabet Med.

[12] Nouri A, Tetreault L, Singh A, Karadimas SK, Fehlings MG (2015). Degenerative Cervical Myelopathy: Epidemiology, Genetics, and Pathogenesis. Spine (Phila Pa 1976).

[13] Faster systematic reviews. Rayyan QCRI.

[14] Dimensions.ai.

[15] Davies BM, Goh S, Yi K, Kuhn I, Kotter MRN (2018). Development and validation of a MEDLINE search filter/hedge for degenerative cervical myelopathy. BMC Med Res Methodol.

[16] Wu Zhen-Kai, Zhao Qing‐hua, Tian Ji‐wei, Qian Yong‐bing, Zhou Yi, Yang Fan, Zhao Li, Porter Daniel (2016). Anterior versus posterior approach for multilevel cervical spondylotic myelopathy. Cochrane Database Syst Rev.

[17] Numpy.

[18] Panda Data Analysis Library.

[19] Choropleth maps in Python. Plotly.

[20] Grodzinski B, Bestwick H, Bhatti F, Durham R, Khan M, Partha Sarathi CI, Teh JQ, Mowforth O, Davies B (2021). Research activity amongst DCM research priorities. Acta Neurochir.

[21] Viergever RF, Hendriks TCC (2016). The 10 largest public and philanthropic funders of health research in the world: what they fund and how they distribute their funds. Health Res Policy Sys.

[22] Government/agency RTWB Research and development expenditure (% of GDP) | Data. The World Bank.

[23] Røttingen J, Regmi S, Eide M, Young AJ, Viergever RF, Årdal C, Guzman J, Edwards D, Matlin SA, Terry RF (2013). Mapping of available health research and development data: what's there, what's missing, and what role is there for a global observatory?. The Lancet.

[24] Johnson & Johnson Medical Devices.

[25] Research. MS Society.

[26] MND Association.

[27] News PAOMRC Charities' funding contributes to UK medical research excellence. Association of Medical Research Charities.

[28] Government/agency R UK Parliament. House of Commons - Health - Minutes of Evidence.

[29] Research projects. Myelopathy.org.

[30] Gillum LA, Gouveia C, Dorsey ER, Pletcher M, Mathers CD, McCulloch CE, Johnston SC (2011). NIH Disease Funding Levels and Burden of Disease. PLoS ONE.

[31] Institute OM (1998). Scientific Opportunities and Public Needs: Improving Priority Setting and Public Input at the National Institutes of Health.

[32] Begum M, Lewison G, Lawler M, Sullivan R (2018). Mapping the European cancer research landscape: An evidence base for national and Pan-European research and funding. Eur J Cancer.

[33] Mowforth OD, Davies BM, Goh S, O'Neill CP, Kotter MRN (2020). Research Inefficiency in Degenerative Cervical Myelopathy: Findings of a Systematic Review on Research Activity Over the Past 20 Years. Global Spine J.

[34] Yin M, Xu C, Ma J, Ye J, Mo W (2020). A Bibliometric Analysis and Visualization of Current Research Trends in the Treatment of Cervical Spondylotic Myelopathy. Global Spine Journal.

[35] Yusuf S, Baden T, Prieto-Godino LL (2013). Bridging the Gap: establishing the necessary infrastructure and knowledge for teaching and research in neuroscience in Africa. Metab Brain Dis.

[36] Beaglehole R, Bonita R (2010). What is global health?. Global Health Action.

[37] Waheed MA, Hasan S, Tan LA, Bosco A, Reinas R, ter Wengel PV, Dennis Hey HW, Aleem IS (2020). Cervical spine pathology and treatment: a global overview. J Spine Surg.

[38] Conalogue DM, Kinn S, Mulligan J, McNeil M (2017). International consultation on long-term global health research priorities, research capacity and research uptake in developing countries. Health Res Policy Sys.

[39] Government/agency RKFF (2019). Kaiser Family Foundation: the US Government and Global Health. Kaiser Family Foundation.

[40] Global health research. NIHR.

[41] CIHR: Global Health Research. CIHR.

[42] Abimbola S, Negin J, Martiniuk A (2017). Charity begins at home in global health research funding. The Lancet Global Health.

[43] (2019). Annual Report of the Chief Medical Officer. Government/agency report: APS Group for the Department of Health and Social Care.

[44] Frenk J, Gómez-Dantés O, Moon S (2014). From sovereignty to solidarity: a renewed concept of global health for an era of complex interdependence. The Lancet.

[45] Badhiwala JH, Ahuja CS, Akbar MA, Witiw CD, Nassiri F, Furlan JC, Curt A, Wilson JR, Fehlings MG (2020). Degenerative cervical myelopathy — update and future directions. Nat Rev Neurol.

[46] Research: increasing value, reducing waste. The Lancet.

[47] Chalmers I, Bracken MB, Djulbegovic B, Garattini S, Grant J, Gülmezoglu AM, Howells DW, Ioannidis JPA, Oliver S (2014). How to increase value and reduce waste when research priorities are set. Lancet.

[48] Chan A, Song F, Vickers A, Jefferson T, Dickersin K, Gøtzsche PC, Krumholz HM, Ghersi D, van der Worp HB (2014). Increasing value and reducing waste: addressing inaccessible research. The Lancet.

[49] Ioannidis JPA, Greenland S, Hlatky MA, Khoury MJ, Macleod MR, Moher D, Schulz KF, Tibshirani R (2014). Increasing value and reducing waste in research design, conduct, and analysis. Lancet.

[50] Davies BM, McHugh M, Elgheriani A, Kolias AG, Tetreault LA, Hutchinson PJA, Fehlings MG, Kotter MRN (2016). Reported Outcome Measures in Degenerative Cervical Myelopathy: A Systematic Review. PLoS One.

[51] Davies BM, McHugh M, Elgheriani A, Kolias AG, Tetreault L, Hutchinson PJA, Fehlings MG, Kotter MRN (2017). The reporting of study and population characteristics in degenerative cervical myelopathy: A systematic review. PLoS One.

[52] KHAN DZ, DAVIES BM, KOTTER MR (2020). Spinal Research — A Field in Need of Standardization. J Rheumatol.

[53] Ahmed S, Hirayama Y, Khan D, Mowforth O, Kotter MR, Davies B (2020). Letter to the Editor: The Need for Standardization of Terminology in Spinal Research. Spine (Phila Pa 1976).

[54] Grodzinski B, Mowforth O, Tetreault LA, Davies BM (2020). Improving the Quality of Systematic Reviews in Spinal Surgery Requires Community-Wide Engagement and Pragmatism. Global Spine Journal.

[55] Khan DZ, Khan MS, Kotter MR, Davies BM (2020). Tackling Research Inefficiency in Degenerative Cervical Myelopathy: Illustrative Review. JMIR Res Protoc.

[56] Herzog C, Hook D, Konkiel S (2020). Dimensions: Bringing down barriers between scientometricians and data. Quantitative Science Studies.

[57] Hook DW, Porter SJ, Herzog C (2018). Dimensions: Building Context for Search and Evaluation. Front. Res. Metr. Anal.

[58] Payne A, Siow A (2003). Does Federal Research Funding Increase University Research Output? The B. E. Journal of Economic Analysis & Policy.

[59] Sandström U (2008). Research quality and diversity of funding: A model for relating research money to output of research. Scientometrics.

[60] Funding, finance, and operations. Universities UK.

[61] Xie Q, Freeman RB (2019). Bigger Than You Thought: China's Contribution to Scientific Publications and Its Impact on the Global Economy. China & World Economy.

